# Supplementary material for: Human-type sialic acid receptors contribute to avian influenza A virus binding and entry by hetero-multivalent interactions
Source: Nat Commun. 2022 Jul 13;13:4054. doi: 10.1038/s41467-022-31840-0 (PMC9279479; doi:10.1038/s41467-022-31840-0)
Supplement: Supplementary file 1 — Supplementary information [file 41467_2022_31840_MOESM1_ESM.pdf]

Supplementary Figures

Human-type sialic acid receptors contribute to avian influenza A virus binding and entry by hetero-multivalent interactions

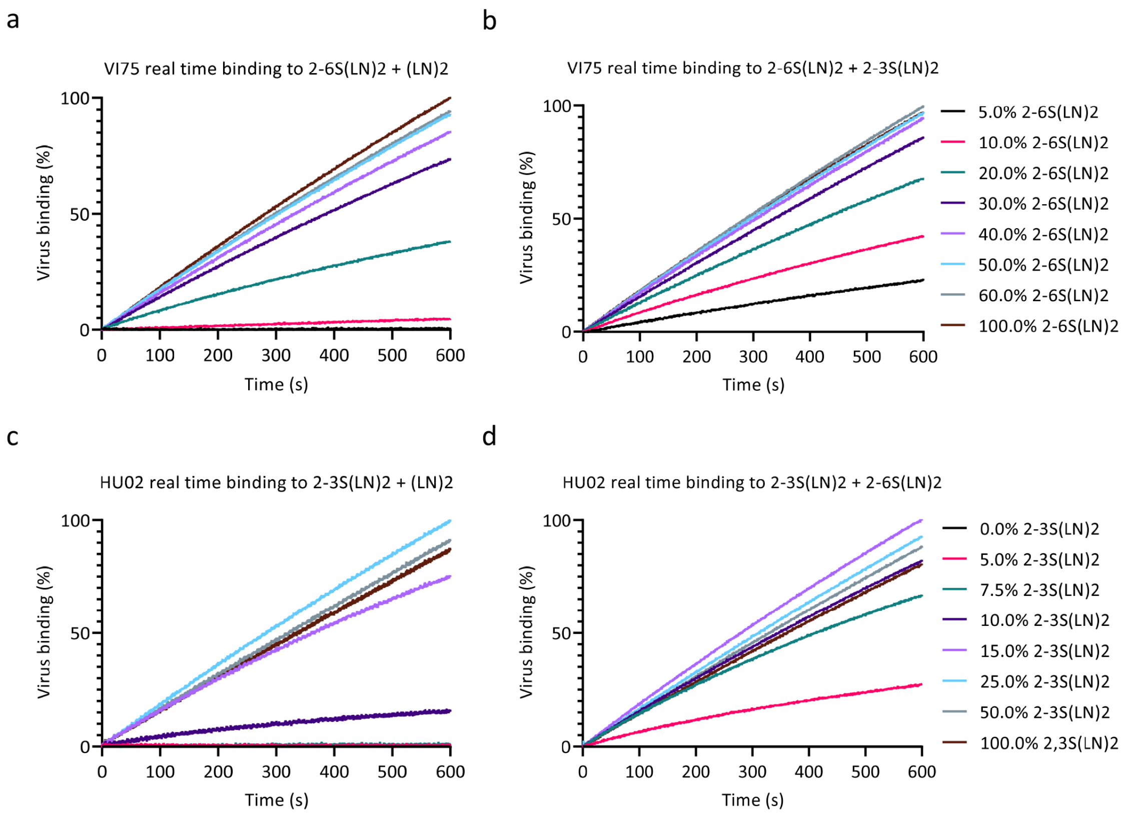

Supplementary Figure 1. **Realtime BLI binding curves for virus strains VI75 and HU02. a-c,** ~100pM suspensions of human H3N2 strain VI75 (**a,b**) or avian H5N1 strain HU02 (**c,d**) were associated to 8 BLI sensors loaded with different relative densities of human-type receptor 2-6S(LN)2 (**a,b**) or avian-type receptor 2-3S(LN)2 (**c,d**) as indicated in the figure. Specific receptors were complemented to 100% density with either the asialoglycan (LN)2 (**a,c**) or the low-affinity receptor 2-3S(LN)2 for VI75 (**c**) or 2-6S(LN)2 for HU02 (**d**). Binding level (y-axis) is shown relative to the highest binding level obtained. The relative initial binding rate is calculated from the slope of the curves during the first 100s and plotted against relative receptor density to obtain the characteristic sigmoidal curves describing initial receptor binding rate as a function of receptor density as shown in Figs. 2 and 3. Note that for HU02 the maximum binding rate is obtained at 25% density of 2-3S(LN)2 and slightly declines at higher receptor density (**c**). For HU02 the maximum binding rate is reached at 100% 2-6S(LN)2. Receptor density at a fully loaded sensor surface (radius of 0.3mm) is 0.0036 receptors per nm^2 (0.6 $pmol/cm^2$) [23]. Source data are provided as a Source Data file.

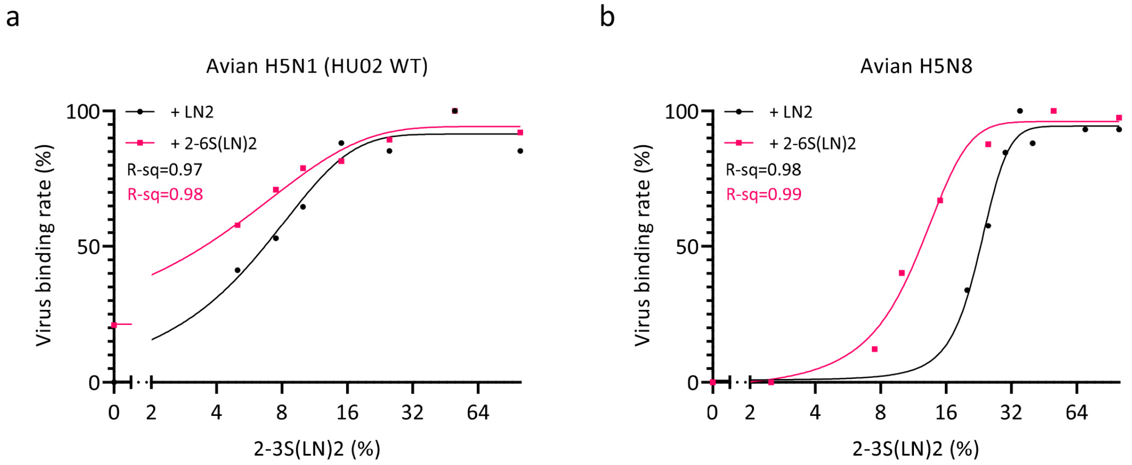

Supplementary Figure 2. Low-affinity receptors lower the threshold density for avian H5N1 and H5N8 IAV binding by heteromultivalent interactions to a heterogenous receptor surface. a,b, Relative virus binding rates were plotted as a function of relative receptor density for H5N1 strains HU02 WT (**a**) and H5N8 (**b**). The relative density of the 2-3S(LN)2 "avian-type" receptor is plotted on the x-axis. The black curves display binding to the 2-3S(LN)2 receptor complemented to 100% density with the asialoglycan LN2 (Galβ1-4GlcNAcβ1-3Galβ1-4GlcNAc) whereas the magenta curves display binding to the 2-3S(LN)2 receptor complemented to 100% density with the 2-6S(LN)2 "human-type" receptor. Relative binding rates are calculated for each point from individual binding curves for each density (see Supplementary Fig. 1 for an example). R-sq values for fitted curves are indicated. Source data are provided as a Source Data file.

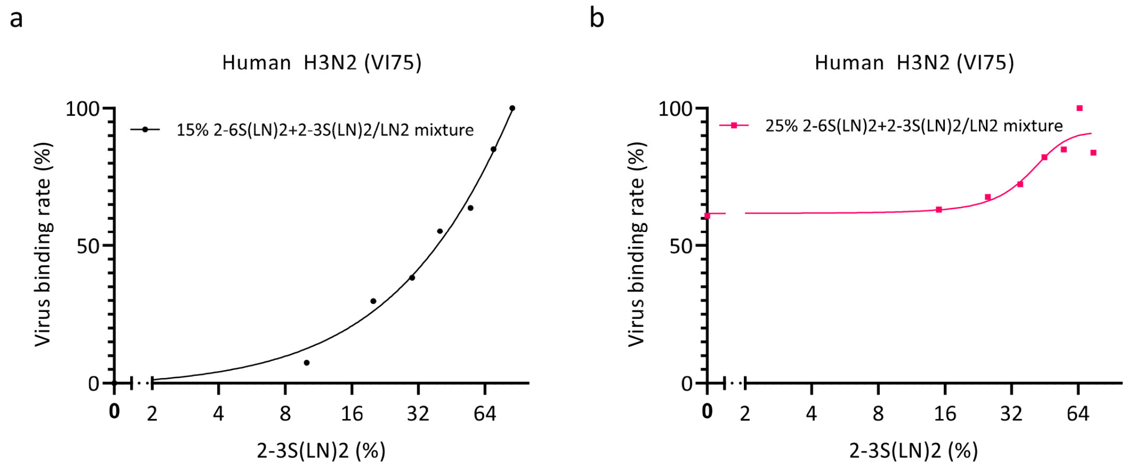

Supplementary Figure 3. Concentration-dependent effect of low-affinity receptor on virus binding rate. a,b, Virus strain VI75 (~100pM) was associated to BLI sensors loaded with 15% (**a**) or 25% (**b**) density of the human-type receptor 2-6S(LN)2 in the presence of increasing densities of the low-affinity avian-type receptor 2-3S(LN)2 and complemented by (LN2). Relative binding rate was plotted as a function of relative 2-3S(LN)2 density (using a 0 to 100% scale, implying that 100% represents a sensor with 15% 2-6S(LN)2+85% 2-3S(LN)2. Source data are provided as a Source Data file.

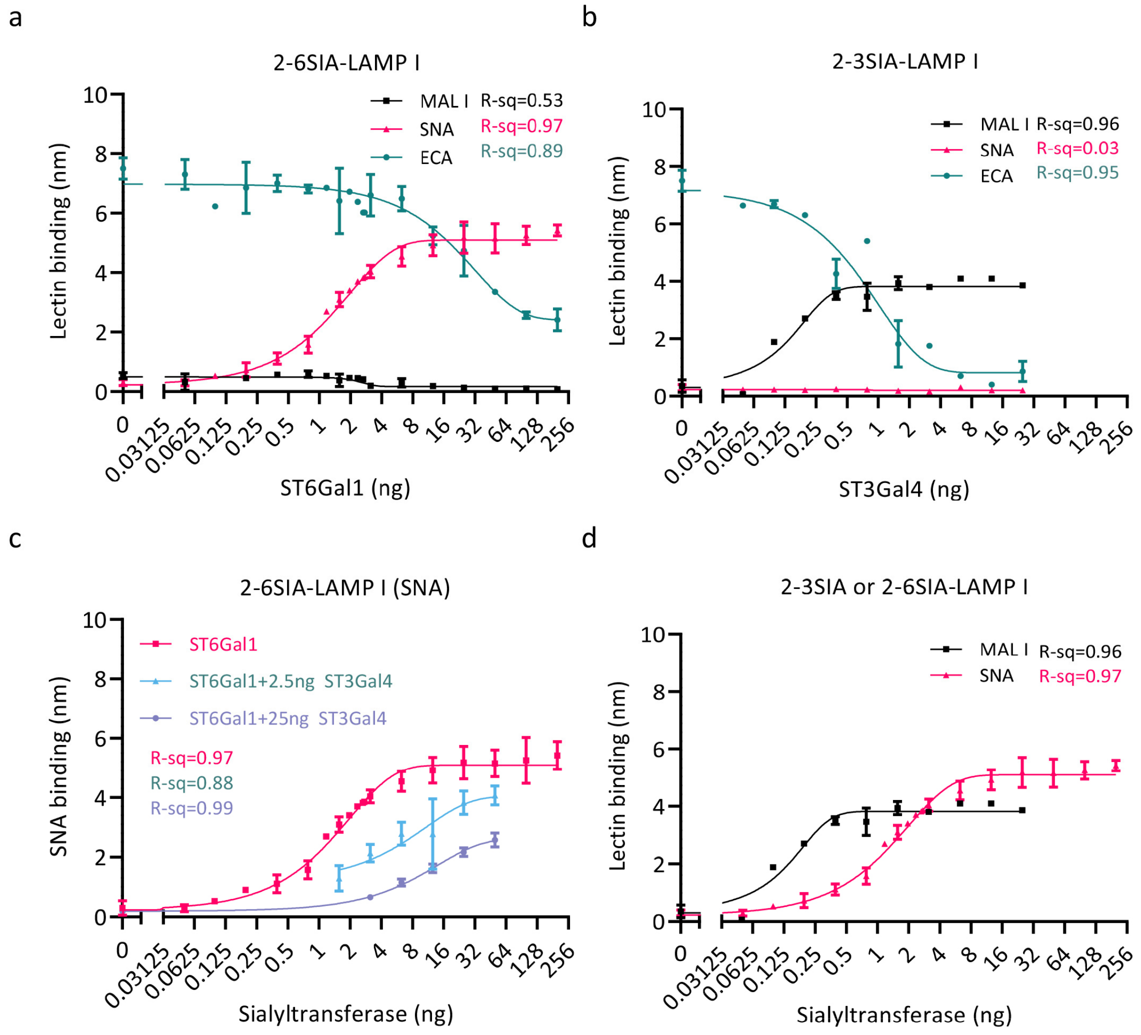

Supplementary Figure 4. Lectin binding to LAMP I produced in HEK$^{\Delta Sia}$ cells co-transfected with increasing amounts of ST6Gal1 or ST3Gal4. BLI sensors were fully loaded with the LAMP I proteins that were produced with increasing amounts (plotted on x-axis) of co-transfected ST6Gal1 (**a**), ST3Gal4 (**b**) or ST6Gal1 plus either 0ng, 2.5ng or 25ng of ST3Gal4 (**c**). (**d**) displays the SNA binding curve of panel a and the MAL I binding curve of panel b in a single diagram to show that increasing ST6Gal1 or ST3Gal4 gives a similar quantitative effect on lectin binding. Sensors were associated with lectins SNA (binding to 2-6Sia), MAL I (binding to 2-3Sia) or ECA (binding to non-sialylated LacNAc termini). Data points were assembled from multiple independent biological replicates (n values are shown in source data). Data are presented as mean values +/- S.D. Note that the ECA binding curve to LAMP I derived from co-transfections with ST6Gal1 (**a**) flattens around 2.5nm indicating that not all LacNAc termini have become sialylated. Source data are provided as a Source Data file.

a

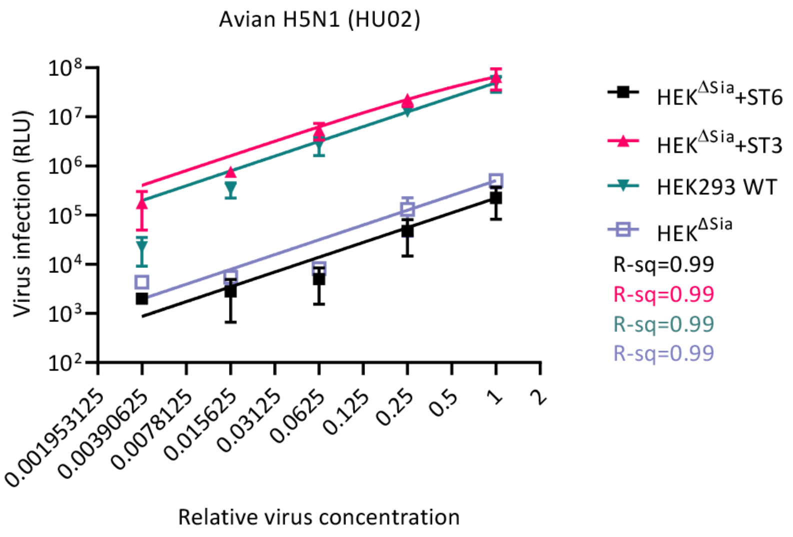

b

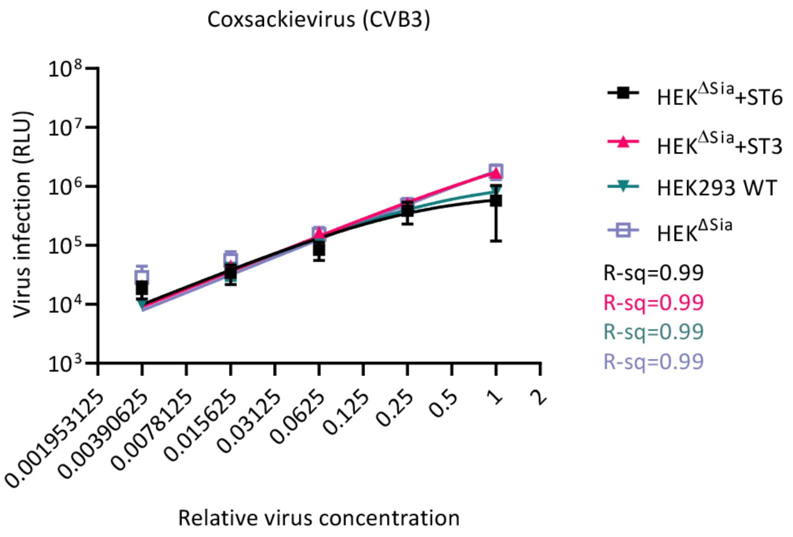

Supplementary Figure. 5. Infection of HEK293 and HEK$^{\Delta Sia}$ **cells by CVB3**. Coxsackievirus B3 uses the coxsackie-adenovirus receptor for cell binding an entry and is independent of Sia receptors. HEK$^{\Delta Sia}$ cells were transfected with 25ng ST6Gal1 (black lines) or 25ng ST3Gal4 (magenta lines) and wildtype HEK293 and HEK$^{\Delta Sia}$ cells were mock-transfected. A single-round infection with 2-3Sia-specific virus strain HU02 (**a**) or CVB3 (**b**) was performed and infection levels were quantified after 17hrs or 8hrs respectively using a luciferase assay. Infections were performed at the virus dilutions plotted on the x-axis. Typical examples of 3 technical triplicates from 2 biological replicates are shown for each virus. R-sq values for fitted curves are indicated. Data are presented as mean values +/- S.D. curves are indicated. Data are presented as mean values +/- S.D. Source data are provided as a Source Data file.

a

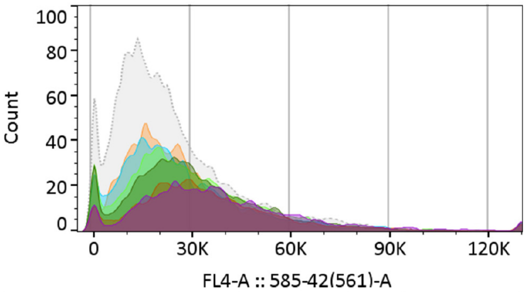

	Sample Name	Subset Name	Count	Geometric Mean: FL4-A
	MALI-0ng ST3Gal4.fcs	*GFP+*	*5031*	*18863*
	MALI-0.37ng ST3Gal4.fcs	GFP+	2858	21181
	MALI-1.1ng ST3Gal4.fcs	GFP+	2794	21704
	MALI-3.3ng ST3Gal4.fcs	GFP+	2737	24372
	MALI-10ng ST3Gal4.fcs	GFP+	2560	25559
	MALI-30ng ST3Gal4.fcs	GFP+	1826	29514
	MALI-90ng ST3Gal4.fcs	GFP+	1927	32281

b

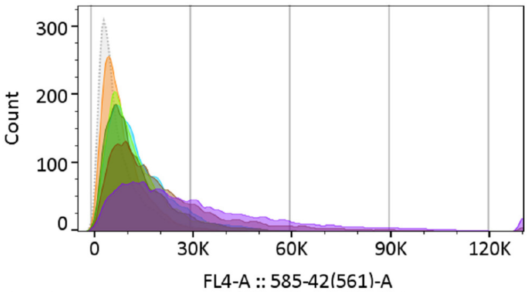

	Sample Name	Subset Name	Count	Geometric Mean: FL4-A
	SNA-0ng ST6Gal1.fcs	*GFP+*	*5167*	*6583*
	SNA-0.19ng ST6Gal1.fcs	GFP+	5411	7619
	SNA-0.56ng ST6Gal1.fcs	GFP+	6112	11875
	SNA-1.67ng ST6Gal1.fcs	GFP+	5712	10844
	SNA-5ng ST6Gal1.fcs	GFP+	5608	11036
	SNA-15ng ST6Gal1.fcs	GFP+	6124	16788
	SNA-45ng ST6Gal1.fcs	GFP+	5556	24531

c

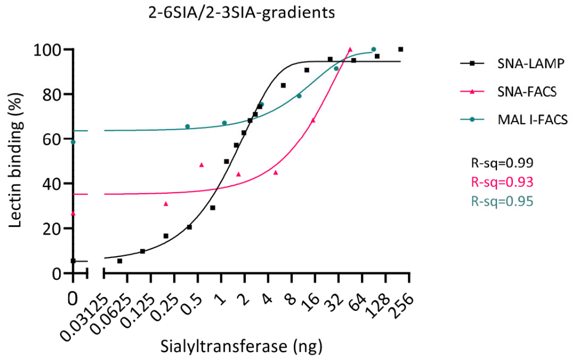

Supplementary Figure. 6. Determination of Sia density on HEK$^{\Delta Sia}$ **cells transfected with sialyltransferases. a-b**, FACS analysis of HEK$^{\Delta Sia}$ cells transfected with ST3Gal4 (2-3Sia) (**a**) or ST6Gal1 (2-6Sia) (**b**) at a concentration range from 0 to 90ng as shown in the color-coded table. Cells were stained (Streptavidin-Alexa568) by incubation with biotinylated lectins MAL I (**a**) or SNA (**b**). Cells were co-transfected with a GFP expression plasmid and GFP+ cells were gated (488nm). X-axis displays fluorescent emission by the 584-42 (561) channel. Y-axis is cell count. **c**, Relative mean fluorescence intensities for MAL I (teal line) and SNA (magenta line) are plotted in parallel to the SNA binding curve to LAMP I of Supplementary Figure. S3 (black line) against the amounts of sialyltransferases used for transfection. Source data are provided as a Source Data file.

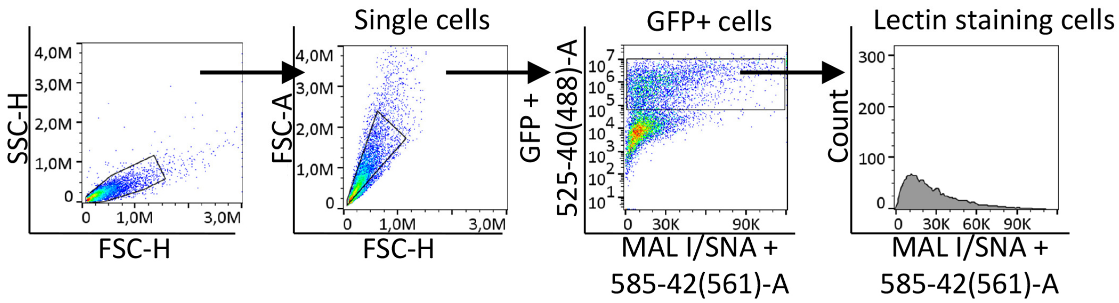

Supplementary Figure 7. Gating strategies for flow cytometric analysis. Representative flow cytometry gating strategy for lectin staining of cells. Related to Fig.S6.
