# Supplementary material for: Human-type sialic acid receptors contribute to avian influenza A virus binding and entry by hetero-multivalent interactions
Source: Nat Commun. 2022 Jul 13;13:4054. doi: 10.1038/s41467-022-31840-0 (PMC9279479; doi:10.1038/s41467-022-31840-0)
Supplement: Supplementary file 3 — Reporting Summary [file 41467_2022_31840_MOESM3_ESM.pdf]

nature portfolio

Corresponding author(s): Erik de Vries, Cornelis A.M. de Haan

Last updated by author(s): Jul 1, 2022

Reporting Summary

Nature Portfolio wishes to improve the reproducibility of the work that we publish. This form provides structure for consistency and transparency in reporting. For further information on Nature Portfolio policies, see our Editorial Policies and the Editorial Policy Checklist.

Statistics

For all statistical analyses, confirm that the following items are present in the figure legend, table legend, main text, or Methods section.

n/a	Confirmed	
☐	☒	The exact sample size (*n*) for each experimental group/condition, given as a discrete number and unit of measurement
☐	☒	A statement on whether measurements were taken from distinct samples or whether the same sample was measured repeatedly
☐	☒	The statistical test(s) used AND whether they are one- or two-sided<br>*Only common tests should be described solely by name; describe more complex techniques in the Methods section.*
☒	☐	A description of all covariates tested
☐	☒	A description of any assumptions or corrections, such as tests of normality and adjustment for multiple comparisons
☐	☒	A full description of the statistical parameters including central tendency (e.g. means) or other basic estimates (e.g. regression coefficient) AND variation (e.g. standard deviation) or associated estimates of uncertainty (e.g. confidence intervals)
☐	☒	For null hypothesis testing, the test statistic (e.g. *F*, *t*, *r*) with confidence intervals, effect sizes, degrees of freedom and *P* value noted<br>*Give P values as exact values whenever suitable.*
☒	☐	For Bayesian analysis, information on the choice of priors and Markov chain Monte Carlo settings
☒	☐	For hierarchical and complex designs, identification of the appropriate level for tests and full reporting of outcomes
☒	☐	Estimates of effect sizes (e.g. Cohen's *d*, Pearson's *r*), indicating how they were calculated

Our web collection on statistics for biologists contains articles on many of the points above.

Software and code

Policy information about availability of computer code

Data collection	ForteBio Data Acquisition (version 9.0.0.26), NanoSight NS3000 (Malvern Panalytical), Renilla Luciferase Assay system (Promega), BC CytoFLEX Flow Cytometer (Beckman Coulter).
Data analysis	ForteBio Data Acquisition (version 9.0.0.26), ForteBio Data Analysis software (version 9.0.0.10), GraphPad Prism 8.4.0, CytExpert for BC CytoFLEX Flow Cytometer Acquisition(Beckman Coulter), FlowJo (version 10), GloMax Discover System GM3000 luminometer (Promega).

For manuscripts utilizing custom algorithms or software that are central to the research but not yet described in published literature, software must be made available to editors and reviewers. We strongly encourage code deposition in a community repository (e.g. GitHub). See the Nature Portfolio guidelines for submitting code & software for further information.

Data

Policy information about availability of data

All manuscripts must include a data availability statement. This statement should provide the following information, where applicable:

- Accession codes, unique identifiers, or web links for publicly available datasets
- A description of any restrictions on data availability
- For clinical datasets or third party data, please ensure that the statement adheres to our policy

Figures have associated raw BLI data. All data needed to evaluate the conclusions in the paper are present in the paper and/or the Supplementary Materials. All raw BLI data that support the findings of this study are available from the corresponding author upon reasonable request.

Field-specific reporting

Please select the one below that is the best fit for your research. If you are not sure, read the appropriate sections before making your selection.

- [x] Life sciences
- [ ] Behavioural & social sciences
- [ ] Ecological, evolutionary & environmental sciences

For a reference copy of the document with all sections, see nature.com/documents/nr-reporting-summary-flat.pdf

Life sciences study design

All studies must disclose on these points even when the disclosure is negative.

Sample size	No statistical methods were used to predetermine sample sizes. The number of required technical replicates and independent biological replicates that were performed was determined, as common in the field, by the requirement for statistical significance.<br>Biolayer interferometry measurements were technically replicated at least 3 times (n=3). Three biological replicates (n=3) were performed for each experiment with similar results. Typical examples were shown or, where indicated, datapoints from different replicates were assembled in to single curves with highly significant curve fitting values (Rsq values indicated in the figures). This procedure is common practice for biolayer interferometry (doi https://doi.org/10.1038/s41467-022-28768-w)<br>For infection experiments, 30.000 cells were taken per condition. For Flow cytometry data, a standard number of 10.000 cells per sample was analyzed.
Data exclusions	No data were excluded from the study.
Replication	Virus infections were guided by prior virus particle number determination to standardize infection dose. No unsuccessful infections did occur. Data were normalized per experiment to account for inevitable small differences potentially due to variations in cell quality and passage number. All infection experiments were successful. Biolayer interferometry profiles obtained from technical replicates always provide nearly identical curves and were therefore successful. Statistical significance for bar diagrams of binding rates derived from BLI data were obtained from three biological replicates. Infection experiments were performed in triplicates within each experiment with at least two biological replicates of all experiments. FACS experiments were biologically replicated twice.
Randomization	For technical and biological replicates samples were usually not applied to the same positions in microtiter plates or BLI sensors. There are also no indications that positioning causes a bias in the measurements. For both BLI an infection randomization is also not possible because pipetting of microtitre plates which harbor many different conditions requires well organized pipetting schemes.
Blinding	Blinding was not relevant as the reported data are based on quantitative measurements.

Reporting for specific materials, systems and methods

We require information from authors about some types of materials, experimental systems and methods used in many studies. Here, indicate whether each material, system or method listed is relevant to your study. If you are not sure if a list item applies to your research, read the appropriate section before selecting a response.

Materials & experimental systems

n/a	Involved in the study	
x		Antibodies
	x	Eukaryotic cell lines
x		Palaeontology and archaeology
x		Animals and other organisms
x		Human research participants
x		Clinical data
x		Dual use research of concern

Methods

n/a	Involved in the study	
x		ChIP-seq
	x	Flow cytometry
x		MRI-based neuroimaging

Eukaryotic cell lines

Policy information about cell lines

Cell line source(s)	HEK293 cells (SIGMA Cat#85120602) were used as described (https://doi.org/10.1016/j.molcel.2019.05.017) in the lab of CB, YN and HC. Construction of the HEKΔSia cell line from these HEK293 cell was described in Büll, C. et al. Probing the binding specificities of human Siglecs by cell-based glycan arrays. Proc Natl Acad Sci U S A 118, e2026102118 (2021); MDCK-II cells were obtained from ATCC (CRL-2936).
Authentication	Cell lines were not authenticated again genetically. HEKΔSia cells of two independent clones were compared during the study and were regularly checked for lack of Sia expression by lectin staining.
Mycoplasma contamination	We confirm that all cell lines were regularly tested negative in mycoplasma tests.
Commonly misidentified lines (See ICLAC register)	Commonly misidentified cell lines were not used.

Flow Cytometry

Plots

Confirm that:

- [x] The axis labels state the marker and fluorochrome used (e.g. CD4-FITC).
- [x] The axis scales are clearly visible. Include numbers along axes only for bottom left plot of group (a 'group' is an analysis of identical markers).
- [x] All plots are contour plots with outliers or pseudocolor plots.
- [x] A numerical value for number of cells or percentage (with statistics) is provided.

Methodology

Sample preparation	HEK293ΔSia cells were seeded in 6-well plates at a density of 1.5E+005 cells/well and 24 hours later cotransfected with a pEGFP reporter plasmid and different amounts and ratios of sialyltransferases (ST6Gal1 and/or ST3Gal4). After 48h transfected cells were released using Cell Dissociation Buffer (Gibco), washed once in phosphate buffered saline (PBS), and fixed with 4% paraformaldehyde (PFA) in PBS. Cells firstly were incubated with biotinylated-lectins (MAL I, Vector Labs; SNA, Vector labs) for 1 hr and then complexed with Streptavidin for 1 hr, (Alexa Fluor™ 568 conjugate (1mg/mL) ,Thermo Fisher). Cells were analyzed on BC Cytoflex LX (Beckman)using CytExpert fot CytoFLEX Acquisition and Analysis software. The gating methods are based on standard protocols.
Instrument	CytoFLEX LX Flow Cytometer, Beckman Coulter
Software	CytExpert Software, FlowJo software V.10
Cell population abundance	Cells were not sorted into fractions. Cell numbers and gating are reported in supplementary figures 6 and 7.
Gating strategy	Populations were gated for single cells based on forward (FSC) and side scatter (SSC). Mock transfection staining was based on GFP positive populations (488nm). Viable positive GFP monocytes were identified based on bitinylated-lectins complexed with Streptavidin Alexa Fluor™ 568 conjugate.

- [x] Tick this box to confirm that a figure exemplifying the gating strategy is provided in the Supplementary Information.
